# Return on Investment of Three-Year Accelerated Programs for Students, Medical Schools, Departments, and Community

**DOI:** 10.1007/s40670-024-02043-7

**Published:** 2024-05-02

**Authors:** Sally A. Santen, Alicia Gonzalez-Flores, Catherine L. Coe, Michael Partin, Judith M. Brenner, Peter M. Nalin, Allison A. Macerollo, Joan Cangiarella, Arthur Saavedra, Shou Ling Leong

**Affiliations:** 1 Department of Emergency Medicine, University of Cincinnati, 231 Albert Sabin Way, ML 0769, Cincinnati, OH 45267-0769 USA; 2 Virginia Commonwealth University School of Medicine, Richmond, VA USA; 3 Department of Internal Medicine, University of California Davis School of Medicine, Davis, CA USA; 4 Department of Family Medicine, University of North Carolina, Chapel Hill, NC USA; 5 Department of Family and Community Medicine, Pennsylvania State University College of Medicine, Hershey, PA USA; 6 New York University Grossman Long Island School of Medicine, Mineola, NY USA; 7 University of Minnesota School of Medicine, Minneapolis, MN USA; 8 Department of Pathology, NYU Grossman School of Medicine, New York, NY USA; 9 Department of Family and Community Medicine, The Ohio State University College of Medicine, Columbus, OH USA; 10 Virginia Commonwealth University, Richmond, VA USA

**Keywords:** Medical school, Residency, Accelerated programs, Innovation

## Abstract

Building on the initial accelerated pathway programs in the 1970s to increase workforce, nearly 30 schools have launched accelerated 3-year pathways (A3YP) during the past decade. The authors based on their educational roles, experiences, and scholarship with A3YP provide this perspective of the argument for A3YP and potential disadvantages for each group—students, schools, residencies, departments, and community. When schools consider innovations, they might consider A3YPs for multiple reasons; this perspective helps provide justification for the program and broadly considers return on investment (ROI). The ROI for students includes decreased debt, reduced costs and stress associated with the fourth-year residency applications, and a directed pathway with facilitated transition into a residency program with accompanying professional identity development. Disadvantages for students include early specialty commitment, risk of deceleration, and condensed curriculum. The ROI for schools includes recruiting and retaining students, who will then transition more easily into residency and stimulating innovation. Residency programs gain residents with known skills, who have been a part of the department for 3 years. In addition, fewer residency slots for interviewing leads to saving recruitment administrative costs and time. Finally, many programs are intended to increase the workforce, since students who come to the region for medical school and transition directly into residency are likely to stay in the region. Disadvantages include increased curricular complexity for the medical school, increased administrative support, and advising resources. Finally, several of the accelerated programs attract matriculants from diverse backgrounds contributing to the diversity of the medical school, residency program, and community workforce.

## Introduction

Building on the initial pilots of developing accelerated pathways in the 1970s to increase workforce, specifically in family medicine and internal medicine [1–6], the move to accelerated 3-year pathway (A3YP) started over two decades ago with recent increased interest [7–13]. At this point, there are over 600 graduates. Based on our collective experiences and scholarship with 3-year accelerated programs, our group of educational leaders provides this perspective in support of accelerated pathways and disadvantages (Tables 1 and 2) for each key group (students, medical schools, residency programs and host departments, and communities) based on our experiences and scholarship. When schools contemplate innovating curricula, they may consider accelerated pathways for all or a subsection of their graduates focusing on one or more specialties for multiple reasons, and this perspective may help provide justification for the return on investment (ROI) from education leaders. We defined broadly to include advantages to different key engaged groups including students, residencies, and medical schools.

**Table 1:** Advantages of accelerated 3-year programs

How do A3YP benefit medical students? 1. Reduce the duration of medical school by 1 year 2. Facilitate the transition from UME to GME 3. Augment specialty identity formation 4. Decrease debt 5. Reduce residency application and away rotation costs 6. Earlier entry into practice by 1 year
How do A3YP benefit medical schools? • Focal point for recruitment • Retain students as future residents • Decrease overall student debt for school • Stimulate educational innovation • Focal point of philanthropy or endowment
How do A3YP benefit residencies and departments? • Optimizes filling in the Match • Gain resident physicians with known skills • Match students already familiar with the GME program • Save program resources by filling positions with A3YP candidates
How do A3YP benefit the community and physician workforce needs? • Optimize the geographic retention of physicians • Augment the diversity of the physician workforce • Alleviate the physician shortage

**Table 2:** Disadvantages: student perspective

Early specialty commitment: students commit upon matriculation to the program leaving less opportunity to changing career decisions.
Risk to decelerate: for students who struggle academically, personally or change specialties, deceleration is difficult and could mean leaving the A3YP entirely.
For some programs, there is a loss of summer breaks. In order to complete the requisite 130 weeks of curricular time as described by the LCME, students may be required to use summers before year 1 and/or between years 1 and 2 of their programs. This could result in less time available to do research and extracurricular activities.
Applying to the Match: because of the timing of the Match coinciding with the student’s 3 years in the program, there is little time to complete rotations outside of one’s home institution. In addition, students from A3YP may appear less competitive than their 4-year counterparts in terms of accomplishments (research, community service, etc.).
Program directors may lack of familiarity with A3YP. As many of these programs are new, program directors have not had experience with many graduates from A3YP. For students entering the Match for residency, the lack of historical perspective could place students at a disadvantage.
Sense of isolation: In schools in which there are 3-year and 4-year tracks, students in the A3YP could feel a sense of isolation from their peers. This could contribute to increased potential for imposter syndrome.
USMLE Step 1 and Step 2 preparation time: students could have less time to prepare for licensing exams.
Less clinical training in the fourth year.

**Fig. 1:**
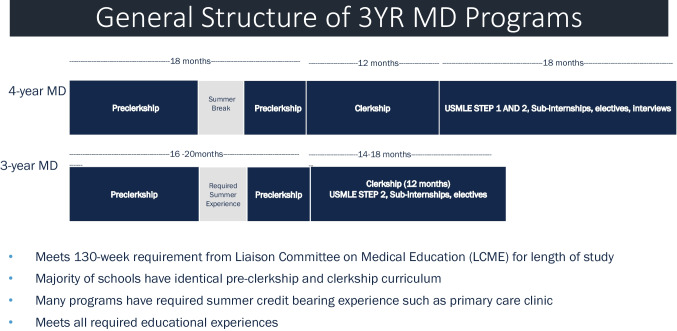
General structure of 3-year MD programs

Medical students enter an A3YP upon matriculation or during the first or second year, depending on the program and complete a minimum of 130 weeks (Fig. 1) [11–13]. Ninety percent of A3YP programs provide medical students a directed pathway into a residency program sponsored by, or affiliated with, their institution. Although a few institutions have an exemption to the National Residency Match Program (NRMP), most medical students enroll and participate in the NRMP in the third year, earn the medical degree (MD) degree in 3 years, and then proceed to residency training. The common form of A3YP is a track in parallel to the 4-year program. While this allows for shared resources, the disadvantage of this model is the logistic complexities of running two programs. Regional campuses and schools have been created where the entire cohort is an A3YP. The advantage is to increase clinical training sites by placing it in communities that welcome partnership in training future physicians in the area. The disadvantages are major frameshift to move 3-year program, difficulty in deceleration, and the requirement for early career selection.

## Student Perspective

There are numerous benefits for medical students. First, the A3YP streamline medical education and facilitate the undergraduate medical education (UME) to graduate medical education (GME) transition. Students have the potential to engage early with their future department’s faculty for career and research mentoring during medical school and across the transition to GME. Consequently, students develop meaningful relationships in the department with faculty and residents and experience less stress over the residency application process. As a result of the UME-to-GME training continuum in the A3YP, students note that they are learning for future practice, and some of the competitive aspects of the clerkship experience may be diminished. While transitioning into residency, the newly graduated doctors are familiar with the learning environment and the residency personnel which leads to a smoother transition. While there is concern for decreased well-being due to the accelerated program, research shows that is not the case based on a study of Association of American Medical Colleges Graduation Questionnaire (AAMC GQ) responses comparing A3YP to standard graduates [14]. The study showed the burnout scores (exhaustion and disengagement) were similar between the accelerated students and those in the 4-year program [14]. The accelerated students rated the learning environment (emotional climate and student-faculty interaction) statistically more positively than the non-accelerated students (*P* < .001) [14]. As students choose their specialty earlier, there may be clearer professional identity formation. The change in purpose has the potential to promote authentic learning experiences and wellness, reduce stress, and assist students on the journey of lifelong learning which we value in medical education. In addition, the cohort of A3YP students form early relationships with like-minded individuals who serve as a support system throughout their accelerated journey. An additional benefit is the support and mentoring they gain from upper classmates and interns or residents who are graduates of their A3YP.

In terms of academics, early studies of 3-year programs demonstrated similar academic outcomes to 4-year programs [1, 2, 5]. Analyses of students’ perceptions of residency readiness using AAMC GQ data found that A3YP students reported feeling as prepared for residency as their 4-year program peers. [14] One study which compared outcomes for 3-year graduates as compared to 4-year graduates showed that students performed similarly in medical school and early residency [15]. Our group has a current study exploring the Accreditation Council for Graduate Medical Education milestones of 3-year compared to 4-year graduates. Carefully crafted off-ramps from the A3YP into the regular 4-year MD program allow medical students flexibility: to change specialty plans, to earn their degree in 4 years, to explore areas of interest and/or earn an additional degree, and or to decelerate for other personal or academic reasons. Students and medical schools may be concerned about deceleration of students from the program. Across our schools, we found transition out of the program was 16% which is similar to medical school attrition.

One of the greatest benefits to a student is the reduction of cost and student debt. Analysis of student debt of A3YP to standard graduates using AAMC GQ data showed that 62.7% of A3YP graduates have less than $100,000 in loans compared to 38.8% of graduates from the 4-year program (*P* < 0.001) [14]. More A3YP graduates reported no medical school debt (41.4%), while only 28% of traditional graduates (*P* < .001) [14].

Students save 1 year of tuition (and the associated loans and interest) and with early entry into practice have an additional year of earnings. We estimate this may be up to $400,000 savings depending on tuition and cost of living. This estimate is based on saving tuition for the fourth year, cost of living for fourth year, residency applications, away rotations, an earlier year of salary, and decreased interest based on earlier loan payments and less debt [16, 17]. As such, decreasing debt and shortening training liberates students to pursue primary care careers. Some programs such as Penn State, Ohio State, and University of California Davis have scholarships for A3YP students.

While the benefits are significant, students note several disadvantages including the need for early specialty selection, condensed training, less clinical training in the fourth year, and decreased time off (Table 2).

## Medical School Perspective

While there is a financial cost to the institution in terms of tuition, faculty time, and administrative support, there are also savings. The A3YP students do not require fourth year clinical rotations and need only minimal career advising, application counseling, or other forms of student affairs and administrative support. As the LCME pays attention to student debt, A3YP markedly decrease the overall student debt.

Many schools found that their A3YP attract students at matriculation who might not have otherwise been interested. The programs build the reputation that the school is innovative and student centric and may create visibility among applicants. As a result, the school may be able to recruit higher caliber students to all programs they offer who may otherwise not attend.

There are additional benefits to these programs. To graduate students early, schools need to be flexible and innovative in the curriculum. Many programs are continuously innovating to improve the program by adjusting the onboarding and bringing in new specialties and programs. Furthermore, several of the A3YP schools received grants due to the novelty of their programs, University of California, Davis (American Medical Association grant), University of North Carolina (American Medical Association grant), New York University Grossman (Macy grant), and Penn State COM (Health Resources & Services Administration grant). From this work, consortium members have presented at number of local, regional, national, and international meetings as well as published [10–13, 15, 16, 18].

A3YP programs can increase diversity. Penn State found that A3YP in the family medicine program increased diversity with 29% of A3YP students from backgrounds underrepresented in medicine. Similarly, University of California, Davis notes the impact of their program on the diversity of the medical school, as 90% of the students in the A3YP come from backgrounds underrepresented in medicine.

Lastly, some programs have been able to leverage philanthropic opportunity based on social mission. Penn State and Ohio State have received endowments for scholarships and the New York University Grossman and New York University Grossman Long Island Schools of Medicine for the entire tuition. In the University of California, Davis, the Accelerated Competency-Based Education in Primary Care (ACE-PC) program contributed to increasing the US News & World Primary Care ranking from 16 in 2014 with 24% of students matching to primary care (PC) in 2014 to 53% (6th) in 2023. At Virginia Commonwealth University School of Medicine, the Competency-Based Graduation program was launched with three goals—decrease the cost of medical school, keep our “best” students for training, and increase the Virginia workforce.

A historical review [6] of accelerated programs a decade ago concluded that A3YPs were initially developed to address physician shortages with significant financial support from government incentives. However, elimination of federal funding, physician shortage decline, and student and faculty dissatisfaction with the compressed nature of the programs led to closure of these initial programs. Despite this history, there is a growth of A3YP, and recent data [14] shows that student satisfaction and wellness is not impacted by accelerated training. Disadvantages to medical schools include the complexity and work of running two programs simultaneously and the loss of tuition dollars. To run an A3YP, schools must provide administrative staff to oversee all aspects of the program from pre-matriculation through graduation, which can place a burden on schools. In addition, schools provide support services to students that are specific to students in the A3YP. In a school that has both three and 4-year programs, this can be challenging. For instance, career planning is different for students in A3YP which means that faculty must be trained accordingly. These additional complexities are ones which every medical school considering the addition of an A3YP must consider.

## Residency Program and Host Department Perspectives

Residency programs gain as well. With the UME-GME continuum, there are opportunities to train and mentor future residents during medical school and to socialize them to the expectations and culture of the department [18]. The handoff between medical school and residency is often dependent on the limited information provided by the MSPE, whereas with A3YP graduates, residency programs are familiar with their A3YP interns’ competencies from day one, while the performance of new interns from other schools is less well known. The opportunities to invite students to seminars, grand rounds, and social celebrations have established relationships both sooner and stronger. Students can get involved in research, community service, and quality improvement collaborations that can extend into residency. Most programs assign students a departmental advisor who can help them navigate the department and shadow in their area of interest. Some programs use A3YP as a recruitment tool to retain the “best” medical school graduates. Other programs use their A3YP to recruit students from diverse backgrounds, which contributes to the diversity of the residency programs. In addition, depending on the number of residency slots assigned to A3YP, students and residency programs can reduce recruiting costs and numbers of interviews. Moreover, students experience a better transition to residency as graduates may be better prepared since they have been engaged in the specialty and department from the beginning of medical school. Many programs have recruited A3YP residents to become chief residents, fellows, or faculty, thus decreasing faculty recruitment costs, which can be quite significant. With residency programs accepting these students into their programs, the communication and bond between UME and GME programs is strengthened. Together they work to design, implement, and recruit high-quality students and ultimately residents.

There are program and department disadvantages including providing time, advising, and experiences that help support students’ acclimation into their departments. Some A3YP students apply and match at programs outside their institution, which can be challenging. A3YP are new and therefore may be met with skepticism by program directors. While early evidence supports students being equally as prepared as 4-year graduates to begin internship, they may lack research and other experiences.

## Community Perspective

One of the great impacts of programs is on the community as A3YP can target workforce development [19]. Studies show that the health of the population is best when 40–50% of the nation’s workforce is made up of PC physicians [20–22]. In the USA, only 37% of the doctors are PC physicians. Some A3YP schools have an explicit social mission with the responsibilities of the healthcare system and medical school to train physicians to serve the healthcare needs of the country. A3YP accelerate the training of the next generation of physicians, in shorter time and at less cost [23]. Of the A3YP who are part of the Consortium of Accelerated Medical Pathway Programs (CAMPP), at least 75% of them have in their mission statement to recruit, mentor, and nurture trainees in specialties that are in shortage, such as primary care, general surgery, psychiatry, and other specialties especially needed in underserved areas.

Mission-specific A3YP are of particular importance for underserved communities. Patients of diverse backgrounds are better served with better outcomes when treated by physicians who are congruent with their background, making the case for increasing the diversity of the workforce [24]. A 2019 AAMC analysis noted that medical students from underrepresented backgrounds are more likely to practice in primary care specialties and to practice in underserved areas [25, 26]. A recent report by the UCSF Healthforce [27] Center found that medical education debt has tripled over the years and that students underrepresented in medicine (UriM) have higher levels of debt compared to non-URIM students. Decreasing the financial burden and removing barriers to pursuing primary care will contribute to diversifying the workforce and ultimately improving the health of underserved communities. Some of the A3YP recruit students from underrepresented and diverse backgrounds including racial/ethnic, socioeconomic, and rural geographic locations. For example, one school found that 57% of students in the Family Medicine Accelerated Pathway expressed a desire to practice in a rural or underserved community.

A3YP that have a direct progression from UME to GME can deliberately partner with GME programs that are placed in highly underserved areas where GME location predicts physician post training practice location. A way to address geographic maldistribution of physicians is by place-specific training, which is facilitated by A3YP programs and their linking of UME to GME.

## Conclusions

We believe these innovative programs can re-engineer the current structure of medical training to provide more efficient ways to train the next generation of physicians for the benefits of both students and patients. There are benefits and disadvantages to address for all key groups. For schools considering programs, they will need to determine whether a small cohort or the entire school or campus will be an A3YP. Further, CAMPP can be a resource [13, 15, 28]. The consortium is engaged to seek evidence of the effectiveness of the programs. A recent study demonstrated recently has shown that accelerated 3-year MD students may perform similarly to 4-year students on ACGME milestones in residency. We are working on an in-depth debt analysis of A3YP versus 4 year graduates. Other work will explore how these graduates perform in medical school and attrition from programs and explore whether they go on to practice in underserved areas.
